# Improved sodium dodecyl sulfate–polyacrylamide gel electrophoresis method for quantifying the 11S/7S ratio in soybean storage proteins

**DOI:** 10.1002/jsfa.70752

**Published:** 2026-06-03

**Authors:** Yaojie Zheng, Cynthia Denbow, Matheus Ogando do Granja, Guillaume Pilot, Zhibo Wang, Maria Luciana Rosso, Bo Zhang

**Affiliations:** ^1^ School of Plant and Environmental Sciences, Virginia Tech Blacksburg VA USA; ^2^ Donald Danforth Plant Science Center Olivette MO USA

**Keywords:** β‐conglycinin, glycinin, protein quantification, 11S/7S ratio, SDS‐PAGE, soybean storage protein

## Abstract

**BACKGROUND:**

Accurate quantification of soybean storage protein subunits glycinin (11S) and β‐conglycinin (7S), and their ratio (11S/7S), is essential for applications in plant breeding, food processing, and allergen research. Although sodium dodecyl sulfate–polyacrylamide gel electrophoresis (SDS‐PAGE) is widely used due to its accessibility and cost‐effectiveness, its quantitative reliability is often limited by variability in protein extraction and band resolution. This study presents an improved SDS‐PAGE protocol optimized for quantifying the 11S/7S ratio with improved accuracy, reproducibility, and cost‐effectiveness.

**RESULTS:**

Two established extraction protocols were evaluated across three soybean cultivars, and a new protocol was developed by integrating their strengths. The new protocol employs a Tris–HCl‐based buffer containing sucrose, ethylenediaminetetraacetic acid, β‐mercaptoethanol, and phenylmethylsulfonyl fluoride, which improves protein solubility and minimizes degradation. Compared to two prior protocols, the new method achieved clearer band resolution, lower error variance, and higher *F*‐values in analysis of variance. Standard curves derived from bovine serum albumin confirmed strong linearity between band intensity and protein quantity (*r* ^***2***^ = 0.993, 0.985), supporting reliable quantitative analysis. Reproducibility tests demonstrated consistent 11S/7S estimation across independent runs. In an application evaluation across 14 breeding lines, the protocol effectively discriminated genotypic variation, with 11S/7S ratio ranging from 1.29 to 2.20 and separation into seven statistically distinct groups, which further validated the protocol's stability and discriminatory power.

**CONCLUSION:**

This optimized SDS‐PAGE method provides a reliable, scalable tool for phenotyping 11S/7S ratios in diverse soybean germplasm, offering a practical balance between resolution, affordability, and throughput for both research and applied use. © 2026 The Author(s). *Journal of the Science of Food and Agriculture* published by John Wiley & Sons Ltd on behalf of Society of Chemical Industry.

## INTRODUCTION

Despite its economic importance and global agricultural dominance, the molecular composition of soybean (*Glycine max* (L.) Merr.) continues to draw interest from both upstream plant breeding and downstream food processing. As a major source of plant‐based protein and oil for human consumption and animal feed, soybeans remain a critical crop worldwide. In 2024, US farmers planted 86.1 million acres of soybeans, yielding an average of 50.7 bushels per acre and producing 4.37 billion bushels in total.^1^ The nutritional and functional properties are largely attributed to their storage proteins, which comprise approximately 700–800 g kg^−1^ of total seed protein. These proteins are categorized by sedimentation coefficients into four main fractions: 2S, 7S (β‐conglycinin), 11S (glycinin), and 15S. Among them, 7S and 11S are the dominant components, accounting for roughly 400 and 300 g kg^−1^ of total soy protein, respectively.^2^ The 7S globulin (β‐conglycinin) is a trimeric protein composed of α′ (~72 kDa), α (~76 kDa), and β (~52 kDa) subunits, stabilized by hydrophobic and electrostatic interactions.^2^, ^3^ In contrast, the 11S globulin (glycinin) forms a hexameric structure of acidic (~35 kDa) and basic (~20 kDa) chains linked by disulfide bonds, giving it a compact and stable conformation.^2^, ^4^ Recent genetic mapping studies have identified loci regulating the 7S α′ and 11S A‐type subunits, providing insight into the genetic basis for subunit variation among genotypes.^3^

The ratio of 11S/7S proteins – glycinin to β‐conglycinin (RGC) – is a key determinant of soy protein functionality, affecting texture, digestibility, allergenicity, and processing quality. A higher RGC is associated with firmer gel textures, improved water‐holding capacity, and modified allergenic potential.^5^, ^6^ For instance, the RGC influences hepatic low‐density cholesterol regulation, with higher glycinin levels linked to improved cholesterol profiles under simulated gastrointestinal digestion.^7^ Allergenicity is primarily attributed to specific subunits within the 7S and 11S fractions, as both β‐conglycinin and glycinin are recognized as major soy allergens.^5^ In addition, processing conditions, such as heat, pH changes, ultrasound, and proteolysis, alter the structural and functional behavior of these proteins, impacting gelation and performance in food systems.^6^, ^8^, ^9^ This relationship is exemplified in tofu production, where the RGC significantly affects texture; higher glycinin content, which increases particulate protein levels, results in firmer curds.^10^

While much research focuses on the genomic basis of soybean protein,^11^, ^12^, ^13^ accurately quantifying the 7S and 11S protein fractions and their ratio (RGC) remains essential for both soybean breeding and food processing. However, existing analytical methods each have their limitations. High‐performance liquid chromatography (HPLC) provides excellent resolution and specificity but requires expensive instrumentation and reagents, making it impractical for routine or large‐scale analyses. Enzyme‐linked immunosorbent assay (ELISA) offers sensitive quantification and is widely used in allergen detection, yet its effectiveness is constrained by antibody specificity and limited adaptability to high‐throughput workflows.^14^ In contrast, sodium dodecyl sulfate–polyacrylamide gel electrophoresis (SDS‐PAGE) is broadly accessible, relatively low cost, and commonly available in standard laboratory settings. It offers a practical compromise between resolution, throughput, and cost. However, SDS‐PAGE generally provides lower resolution than HPLC and can suffer from limited reproducibility or quantification accuracy, especially when distinguishing proteins with similar molecular weights or across genetically diverse lines.^15^

These tradeoffs underscore the need to develop a standardized, reproducible, and cost‐effective SDS‐PAGE protocol specifically optimized for soybean storage protein profiling. Recent research has shown that the optimization of gel concentration, buffer composition, and sample preparation can significantly enhance the resolution and reproducibility of SDS‐PAGE while maintaining its affordability and simplicity for routine procedures.^15^ Such methodological improvements would benefit both breeding programs and food science applications by enabling more precise protein subunit characterization, reliable quantification of RGC, and consistent quality control across diverse samples.

Given these challenges, this study aims to evaluate two established SDS‐PAGE‐based extraction protocols, develop a modified protocol to enhance resolution and quantitative stability, and validate the method across multiple soybean breeding lines and biological replicates. By addressing these objectives, we seek to advance protein profiling methodologies and support the development of soybean varieties with optimized functional and nutritional attributes.

## MATERIALS AND METHODS

### Plant materials for protocol development

The primary seed materials used for creating the protocol were from three released soybean cultivars: ‘MFL‐5P59’, ‘Glenn’, and ‘VT Barrack’. For the expanded application experiment, a further 14 soybean breeding lines were included. For each soybean genotype, 10 g of clean seeds were sampled. The seeds were ground into a fine, homogeneous powder using a Knifetec 1095 sample mill (Foss Tecator, Hillerød, Denmark) at 20 000 rpm for 10 s. Seed powders were then collected in sealed plastic bags and stored in envelopes at room temperature for further use.

### Defatting and protein extraction

Seed powder weighing 10–20 mg was measured and placed in a 1.5 mL microcentrifuge tube with each genotype sampled four times to form four replications for further experiments. 500 μL defatting solution, composed of chloroform–hexane–methanol in an 8:5:2 (v/v/v) ratio, was added to each tube. The tubes were capped tightly and vortexed for 60 s to thoroughly suspend the powder in the solution. After 1 h standing at room temperature, all samples were centrifuged for 8 min at 10 845 × *g* and 4 °C. The supernatants were removed, and the pellets were resuspended in a fresh defatting solution. This defatting step was repeated twice more. The soybean meal pellet was dried overnight under a fume hood.

Protein extraction buffers from a published protocol^3^ (named Protocol 1) and a protocol provided by Dr Pilot (Virginia Tech, named Protocol 2) were tested. Based on Protocols 1 and 2, a new buffer system (named New Protocol) balancing efficiency, simplicity, and stability was developed. The compositions of the three protocols' buffer systems are presented in Table 1.

**Table 1:** Buffer composition of Protocols 1, 2 and New Protocol

Extraction buffer	Composition
Protocol 1	10 mmol L^−1^ Tris–HCl, pH 8.0; 25 g L^−1^ SDS; 10 mmol L^−1^ β‐mercaptoethanol
Protocol 2	50 mmol L^−1^ potassium phosphate buffer, pH 7.5; 500 mmol L^−1^ sucrose; 3 mmol L^−1^ EDTA; 10 mmol L^−1^ DTT; 1 mmol L^−1^ PMSF
New Protocol	10 mmol L^−1^ Tris–HCl, pH 8.0; 500 mmol L^−1^ sucrose; 3 mmol L^−1^ EDTA; 10 mmol L^−1^ β‐mercaptoethanol; 1 mmol L^−1^ PMSF

SDS, sodium dodecyl sulfate; EDTA, ethylenediaminetetraacetic acid; DTT, dithiothreitol; PMSF, phenylmethylsulfonyl fluoride.

For each protocol, 500 μL of the protein extraction buffer was added to microcentrifuge tubes and vortexed to resuspend soybean meal pellets. The samples were then incubated in ice for 1 h, and vortexed every 15 min to aid the extraction process. Samples were then centrifuged at 15 000 × *g* and 4 °C for 20 min. The supernatants containing soybean protein isolates (SPI) were transferred to a new 1.5 mL microcentrifuge tube.

### Protein subunit quantification

An SDS‐PAGE protocol, based on the NuPAGE Bis‐Tris Mini Gels system (Thermo Fisher Scientific, Waltham, MA, USA) with modifications, was used for profiling soy protein subunits. The sample solution (10 μL total) included 3.5 μL SPI, 2.5 μL NuPAGE LDS Sample Buffer (4×), 1 μL NuPAGE Sample Reducing Agent (10×), and 3.5 μL deionized (DI) water. The sample solution was incubated at 70 °C for 10 min and then loaded on 4–12% NuPAGE Bis‐Tris Mini Protein Gel. Before loading samples, the gel was placed in an XCell SureLock Mini‐Cell (Thermo Fisher Scientific) gel tank with 1× NuPAGE MES SDS Running Buffer; 500 μL NuPAGE Antioxidant was added to the inner buffer chamber to maintain reducing conditions. The gel was run at constant 200 V (85–100 mA) for 40 min to ensure that the protein subunits were properly separated. The commercial precast gel system (NuPAGE Bis‐Tris Mini Gels) was used in this study to ensure consistent electrophoretic conditions and minimize variability associated with gel preparation during method development. Evaluation of the protocol under alternative SDS‐PAGE systems may require additional optimization.

The gel was then stained using SYPRO Protein Gel Stain (Thermo Fisher Scientific). The staining solution was prepared by diluting the stock reagent with 75 mL L^−1^ acetic acid to 0.2 mL L^−1^. 150 mL staining solution was poured into the gel in an appropriately sized container. The lid of the container was securely closed to avoid solution spilling, and the whole container was covered by aluminum foil to avoid direct light. The gel box was shaken for 60 min on a shaker, and the staining solution was replaced with 150 mL of 75 mL L^−1^ acetic acid for a further 60 min under shaking for background removal. The gel was finally gently rinsed with DI water to remove the excess acetic acid.

The gel was imaged by using an Azure 600 imaging system (Azure Biosystems, Dublin, CA, USA) with an excitation wavelength of 472 nm and an emission wavelength of 684 nm. The gel image was downloaded and transferred to Bio‐Rad Image Lab software version 6.1.0 (Bio‐Rad Laboratories Inc., Hercules, CA, USA) for quantifying protein subunits. In this study, the PageRuler Unstained Broad Range Protein Ladder (Thermo Fisher Scientific) was used as the molecular weight standard to identify target protein subunits. Referring to the molecular weight of subunits of glycinin and β‐conglycinin published previously,^16^, ^17^, ^18^ the β‐conglycinin was profiled into three band clusters in this study: α, α′, and β subunits; the glycinin was profiled into four band clusters: A3, A1 + A2 + A4, B1 + B2 + B3 + B4, and A5 subunits. Target bands were annotated in Image Lab software, and their relative fluorescence intensities were extracted. RGC was calculated from the sum of the relative fluorescence intensity of glycinin subunits divided by the sum of the relative fluorescence intensity of β‐conglycinin subunits. For the new protocol, the whole experiment was replicated a second time to compare the results to test its accuracy.

### Application experiment

The protocol's accuracy and discriminatory power were further evaluated using 14 F_4:6_ soybean breeding lines. This experiment used the same defatting, protein extraction, and SDS‐PAGE procedures as above, but was performed in duplicate, given the high repeatability observed in the development study. A Pierce BSA standard (Thermo Fisher Scientific) was prepared and loaded at 6, 3, and 1.5 μg per lane. These standards were used to generate a calibration curve for accurate quantification of glycinin and β‐conglycinin subunits.

### Statistical analysis

In this study, the standard curves were generated in Bio‐Rad Image Lab, and the band density data were exported as a table. For the protocol‐development experiment, genotypic and replication effects were evaluated with a two‐way analysis of variance (ANOVA) using the Fit Model function in JMP Pro 18.0.2 (SAS Institute Inc., Cary, NC, USA). Two‐time technical replicates were compared across cultivars to test repeatability. Multiple comparisons and result visualizations were performed in JMP Pro 18 using the Fit Model function and Graph Builder. For the application experiment, ANOVA was conducted to estimate genotypic effect and replication effect, and Fisher's least significant difference (LSD) was calculated to test significant difference between breeding lines and between replications. Results of the application experiment were also visualized using JMP Graph Builder.

## RESULTS

### Comparisons between protocols

**Figure 1:**
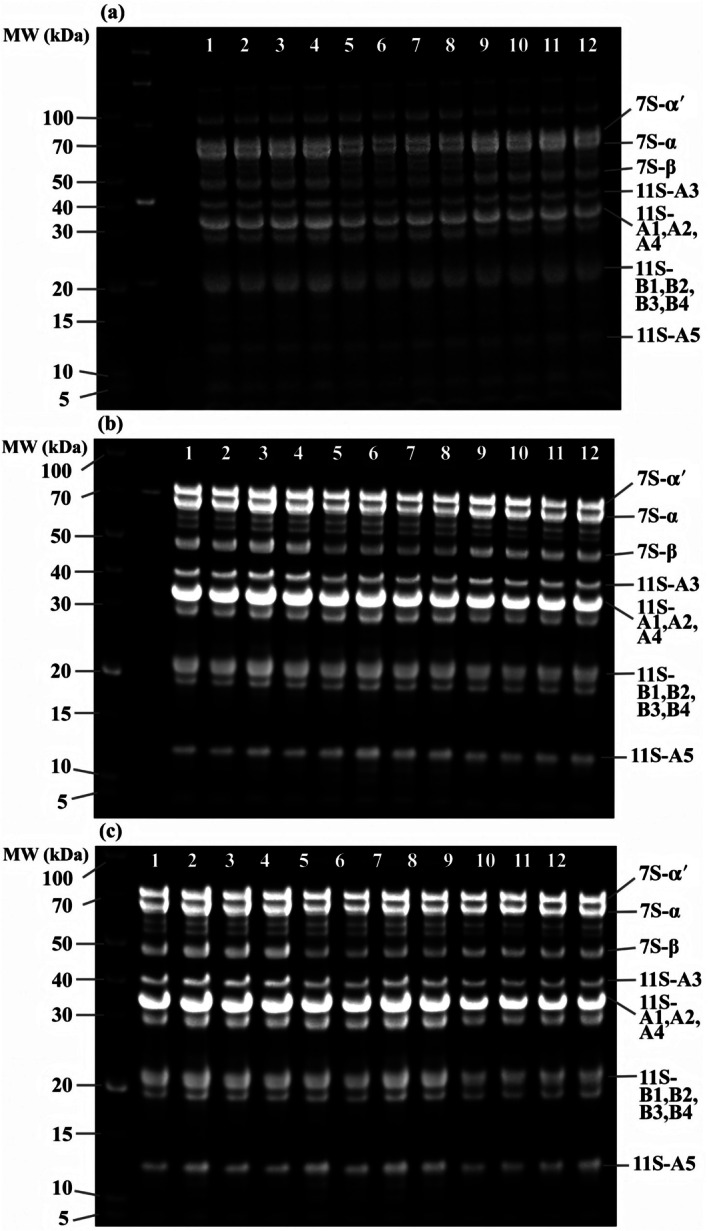
Sodium dodecyl sulfate–polyacrylamide gel electrophoresis images of Protocol 1 (a), Protocol 2 (b), and New Protocol (c). Proteins were separated on 4–12% gradient Bis‐Tris gels (NuPAGE system), stained with SYPRO Protein Gel Stain, and imaged using an Azure 600 imaging system (excitation 472 nm, emission 684 nm). Lanes 1–4 correspond to four replicates of MFL‐5P59, lanes 5–8 to Glenn, and lanes 9–12 to VT Barrack.

In the protocol development experiment, two established protocols (Protocols 1 and 2) were tested, and a new protocol (New Protocol) was developed. The SDS‐PAGE gel images are shown in Fig. 1. Protocol 1 (Fig. 1(a)) produced more smeared bands relative to Protocol 2 (Fig. 1(b)) and the New Protocol (Fig. 1(c)), increasing the likelihood of variability among within‐genotype replicates. Conversely, Protocol 2 and New Protocol showed clearer and sharper bands and were nearly identical, supporting more stable and accurate quantification.

These observations of gel images were further supported by assessing genotype and replicate effects (Table 2). Across all three protocols, the genotypic effect was significant, confirming that each protocol detected differences in the RGC among cultivars. Replicate effects were not significant, indicating good within‐genotype repeatability of the RGC measurement. Nevertheless, Protocol 1 underperformed: its lower genotype *F*‐statistic (*F* = 18.93) and higher error variance align with the smeared bands. Protocol 2 and the New Protocol showed stronger discrimination and precision, reflected in higher *F*‐statistics and lower error mean squares. The New Protocol performed best overall (*F* = 160.40; MSE = 0.0010), outperforming Protocol 2 (*F* = 42.41; MSE = 0.0033) in both discrimination and stability.

**Table 2:** ANOVA results for genotype and replication effects of Protocol 1, Protocol 2, and New Protocol

Protocol	SOV	DF	SS	MS	*F*	Prob. > *F*
Protocol 1	Genotype	2	0.3265	0.1633	18.9296	0.0026
	Replication	3	0.0057	0.0019	0.2207	0.8787
	Error	6	0.0517	0.0086		
Protocol 2	Genotype	2	0.2830	0.1415	42.4139	0.0003
	Replication	3	0.0141	0.0047	1.4068	0.3295
	Error	6	0.0200	0.0033		
New Protocol	Genotype	2	0.3122	0.1561	160.4017	<0.0001
	Replication	3	0.0126	0.0042	4.2993	0.0611
	Error	6	0.0058	0.0010		

SOV, source of variation; DF, degrees of freedom; SS, sum of squares; MS, mean square; *F*, *F*‐statistic; Prob. > *F*, *P*‐value.

### Reproducibility of the new protocol

**Figure 2:**
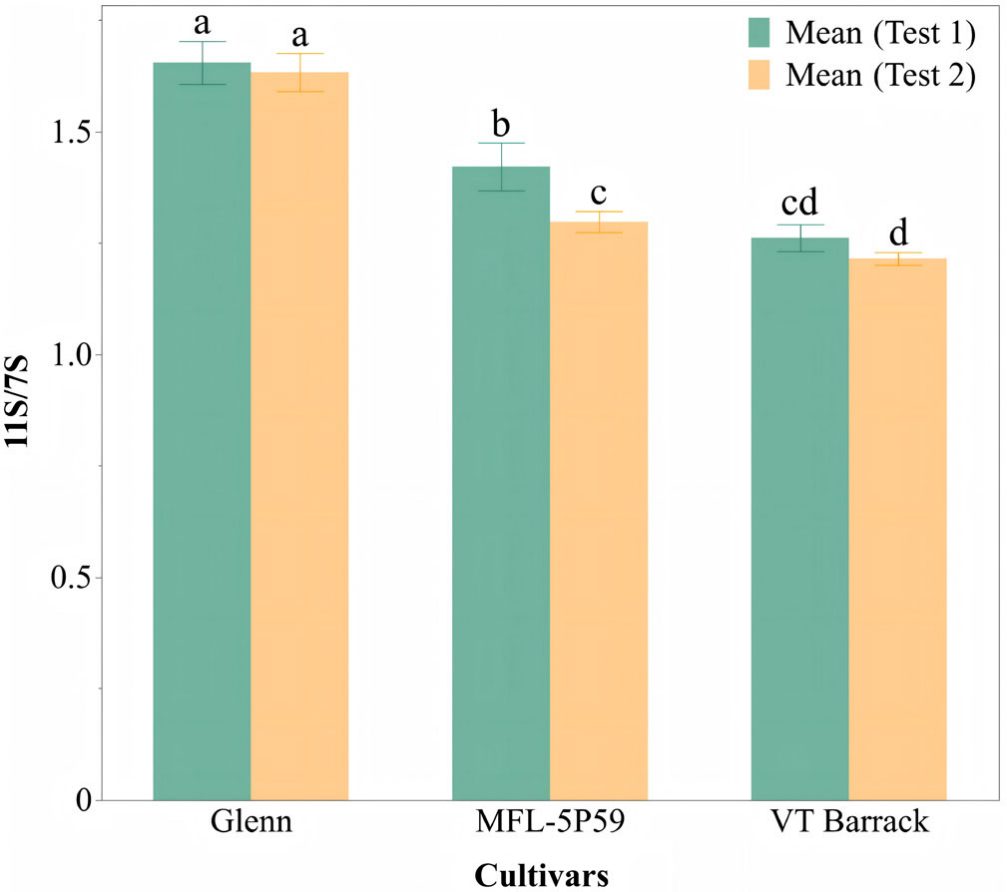
Results of two independent runs of the New Protocol. Proteins were separated on 4–12% gradient Bis‐Tris gels (NuPAGE system), stained with SYPRO Protein Gel Stain, imaged using an Azure 600 imaging system (excitation 472 nm, emission 684 nm), and quantified by densitometric analysis. Bars represent mean values (*n* = 4 per bar); letters above bars indicate significant difference based on least significant difference test (*P* < 0.05); error bars indicate standard deviation.

To further explore the variation between cultivars, a multiple comparison after *F*‐test was conducted (Fig. 2). The results indicated that Glenn had the highest RGC (1.65), followed by MFL‐5P59 (1.42), and VT Barrack showed the lowest ratio (1.26). All RGC differences between cultivars were significant. To further assess the reproducibility of New Protocol, we repeated the workflow from the defatting step (Test 2). The second run reproduced the same pattern of RGC differences among cultivars, indicating good repeatability. Moreover, for Glenn and VT Barrack, the RGC values did not differ significantly between runs, further supporting the protocol's stability and accuracy.

### Application test

**Figure 3:**
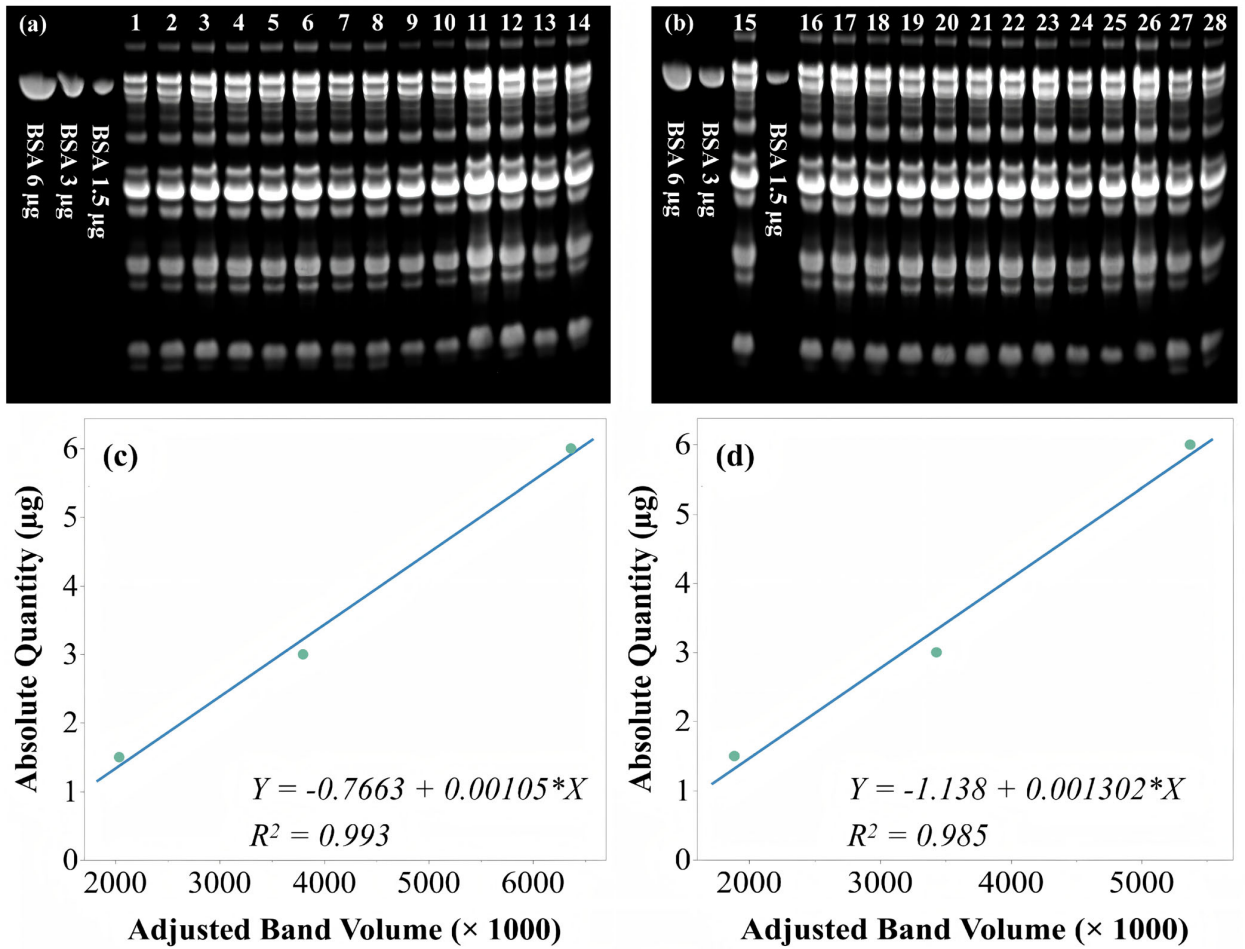
Gel images of 14 breeding lines with bovine serum albumin (BSA) standards (a, b), and standard curves (c, d). Proteins were separated on 4–12% gradient Bis‐Tris gels (NuPAGE system), stained with SYPRO Protein Gel Stain, and imaged using an Azure 600 imaging system (excitation 472 nm, emission 684 nm). Panels (a) and (b) show gel images from two independent gels, with BSA standards (6, 3, and 1.5 μg per lane) and every two consecutive lanes (1–28) containing two replicates of a single soybean breeding line. Panels (c) and (d) show standard curves generated from BSA band intensities for each gel.

To test the broader adaptability of the new protocol in screening more diverse breeding material, we analyzed 14 soybean lines. Samples were run on two gels, with BSA standards loaded at 6, 3, and 1.5 μg per lane (Fig. 3(a),(b)). Standard curves derived from these ladders showed strong linearity between band volume (fluorescence intensity) and protein amount, with *r* ^2^ of 0.99 (gel 1) and 0.98 (gel 2) (Fig. 3(c),(d)). These results indicate that standardized, unbiased protein quantification can be achieved consistently across multiple gels.

Both gels produced clear, well‐resolved bands with good reproducibility, and these observations were further confirmed by ANOVA (Table 3). The genotypic effect was highly significant (*F*‐statistic with *P* < 0.01), indicating that between‐line variation accounted for most of the total variance and that the New Protocol retained strong discriminatory power in a more diverse panel. The replicate effect was significant under the two‐replicate design (*P* = 0.03), consistent with the expected decrease in stability at lower replication; this can be mitigated by increasing the number of replicates. Notably, residual (error) variance remained low in the application test – lower even than that observed for Protocols 1 and 2 in the development experiment.

**Table 3:** ANOVA results of the application experiment of New Protocol

SOV	DF	SS	MS	*F*	Prob. > *F*
Genotype	13	0.9905	0.0762	47.4004	<0.0001
Replication	1	0.0091	0.0091	5.6841	0.0331
Error	13	0.0209	0.0016		

SOV, source of variation; DF, degrees of freedom; SS, sum of squares; MS, mean square; *F*, *F*‐statistic; Prob > *F*, *P*‐value.

**Figure 4:**
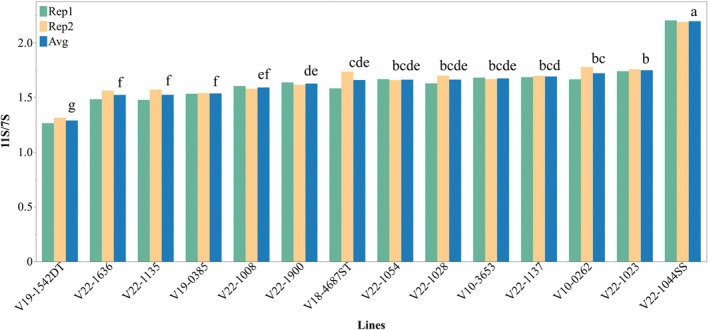
Side‐by‐side bar graph of two replications (Rep1 and 2) and average (Avg) of 14 soybean lines. Proteins were separated on 4–12% gradient Bis‐Tris gels (NuPAGE system), stained with SYPRO Protein Gel Stain, imaged using an Azure 600 imaging system (excitation 472 nm, emission 684 nm), and quantified by densitometric analysis. Bars represent individual replicate values (Rep1 and Rep2; *n* = 1 per bar) and their mean (Avg) for each line. Letters above bars indicate significant differences between the averages, based on least significant difference test (*P* < 0.05).

Following the ANOVA test, we generated a side‐by‐side bar graph to visualize the replicate differences of each line (Fig. 4). Only 5 out of 14 lines (V22‐1636, V22‐1135, V18‐4687ST, V22‐1028, and V10‐0262) showed noticeable replicate differences, indicating that the significant replicate effect was driven by a small subset of lines. Fisher's LSD multiple comparisons were then used to visualize between‐line differences. The RGCs were between 1.29 and 2.20 across the 14 lines, separating into seven significance groups. These results indicate a broad diversity even just for those 14 lines and further confirmed that the new protocol discriminates diverse lines effectively by RGC content.

## DISCUSSION

In this study we developed a new protein extraction protocol by integrating and modifying elements from two existing methods. All three protocols detected genotypic differences in the RGC. However, relative to Protocol 2 and the New Protocol, Protocol 1 produced smeared bands, higher error variance, and a lower genotypic *F*‐statistic, indicating inferior resolution and precision. Although Protocol 1 employs a simple, effective buffer system, its performance was the weakest, likely for three reasons tied to buffer composition.

First, Protocol 1 lacked components that limit protein aggregation during extraction. In contrast, Protocol 2 and New Protocol included sucrose, a non‐reducing sugar that modulates osmotic balance and increases solution viscosity, helping maintain solubility and reduce aggregation during handling.^19^ Second, Protocol 1 lacks broad‐spectrum protease inhibitors. In contrast, Protocol 2 and New Protocol includes ethylenediaminetetraacetic acid (EDTA), which chelates divalent metal ions required by many proteases for activity,^20^ and phenylmethylsulfonyl fluoride (PMSF), a serine protease inhibitor that can inactivate enzymes like trypsin and chymotrypsin.^21^ Their absence likely permitted greater proteolysis, contributing to band smearing and variability. Third, Protocol 1 uses SDS at the solubilization step to denature proteins but lacked stabilizers and protease inhibitors to prevent subsequent degradation and aggregation. While SDS is essential for SDS–PAGE, early exposure without stabilization can unfold proteins, expose cleavage sites, and promote aggregation;^22^, ^23^ a relatively high SDS concentration (25 g L^−1^) in the extract may also interact with the SDS in the running buffer of electrophoresis, affecting migration.

The New Protocol is built based on Protocol 2 but substitutes the buffer and reducing agent (from Protocol 1) to further improve discrimination and stability. Switching from 50 mmol L^−1^ potassium phosphate (pH 7.5) to 10 mmol L^−1^ Tris–HCl (pH 8.0) provides more effective buffering across pH 7.0–9.0, increases globulin solubility without denaturation,^24^, ^25^ and reduces the likelihood of salt‐induced precipitation associated with multivalent phosphate anions.^26^ Tris also matches the chemistry of standard SDS‐PAGE running/stocking buffers and, at lower ionic strength, maintains more uniform conductivity and field strength during electrophoresis, contributing to sharper band resolution and reduced lane‐to‐lane variability. In addition, the Tris system avoids phosphate carryover that can chelate with cations in sample matrices and subtly alter protein mobility.^26^

Replacing 10 mmol L^−1^ dithiothreitol (DTT) with 10 mmol L^−1^ β‐mercaptoethanol (BME; smaller molecular size) may improve access to buried disulfide bonds in complex or tightly folded storage proteins (e.g., 11S glycinin);^27^ although DTT is the stronger, faster reductant, BME's slower kinetics may yield more uniform reduction across batch‐prepared samples. From a practical standpoint, BME and Tris–HCl are simpler to prepare and store, less sensitive to oxidation than DTT solutions, and more cost effective than phosphate/DTT systems – advantages that increase throughput and reduce day‐to‐day drift in phenotyping pipelines.^27^, ^28^ These changes operate alongside the stabilizers and protease inhibitors retained from Protocol 2 – for example, sucrose to limit aggregation and maintain solubility,^19^ EDTA to chelate divalent cations required by many proteases,^20^ and PMSF to inhibit serine proteases such as trypsin and chymotrypsin^21^ – which together reduce proteolysis and aggregation during extraction and storage.

Notably, this study was developed using soybean seed powder as the starting material, and the protein extraction protocol for seed‐based samples represents the primary methodological advance. Application of the workflow to processed soy ingredients (e.g., flours, concentrates, or isolates), which have already undergone protein extraction, requires additional consideration. Prior processing may introduce structural changes such as aggregation, precipitation, or degradation, which can affect band clarity and quantification consistency, as discussed above. Therefore, preliminary testing and matrix‐specific optimization are recommended when adapting the protocol to such systems.

SDS‐PAGE, chromatography‐based methods (e.g., HPLC and reversed‐phase ultra‐performance liquid chromatography), and immunoassays are the three main approaches used to quantify glycinin, β‐conglycinin, and RGC. Among them, SDS‐PAGE is often considered a semi‐quantitative method, as it relies on band intensity, and concerns have been raised about reproducibility across gels.^29^ However, in this study, standard curves generated using BSA standards showed strong linear relationships between band intensity and absolute protein quantity (*r* ^2^ = 0.99 and 0.98), comparable to those reported in ELISA and HPLC studies.^30^, ^31^, ^32^ While RGC calculation does not require absolute quantification, this result suggested the protocol could support it if needed. More importantly, SDS‐PAGE demonstrated high stability in evaluating RGC, with intra‐genotype relative standard deviations (RSD) ranging from 1.1% to 3.7%, comparable with ELISA (~6% RSD) and HPLC (~10% RSD) calculated from recovery rate.^30^, ^31^, ^32^ This improved consistency may come from directly extracting and separating total protein by molecular weight, eliminating dependence on binding kinetics, such as antibody–antigen interactions (ELISA) or enzymatic digestion (HPLC). These other methods also require stricter pH control and are more sensitive to environmental variation, contributing to greater procedural variability.

Although SDS‐PAGE is a conventional method for quantifying soybean protein subunits and their ratios, continued improvements in extraction and electrophoresis steps have made it more reliable. Its simplicity, ease of handling, and low‐cost equipment and reagents make it particularly attractive. In this study, we optimized the protein extraction protocol by integrating and refining two existing methods, resulting in greater stability and repeatability in RGC quantification. The performance achieved was comparable to other mainstream techniques. This method also increases throughput, as it balances technical robustness with simplified reagent preparation and storage. This improved protocol offers a practical phenotyping tool for applications in plant breeding, food processing, and allergen‐related research.

## CONCLUSIONS

This study presents a modified SDS‐PAGE‐based protocol for quantifying the RGC in soybean storage proteins with improved clarity, repeatability, and throughput. By integrating elements from two existing protocols, the newly developed extraction method demonstrates enhanced resolution and stability, as evidenced by clearer gel band profiles, higher *F*‐values, lower error variance, and strong reproducibility across biological replicates and diverse soybean lines. The inclusion of stabilizers, protease inhibitors, and optimized buffer and reducing agents contributes to the protocol's superior performance. Furthermore, the incorporation of a BSA‐based standard curve confirms the method's linearity and semi‐quantitative reliability, offering potential for absolute quantification if needed. The protocol was successfully validated in an expanded set of breeding lines, showing sufficient discrimination power to detect genetic variation in the RGC. With its balance between precision, cost efficiency, and ease of preparation, this medium‐throughput protocol provides a robust tool for protein phenotyping in both breeding and food science research. Future applications may include large‐scale screening for nutritional quality, allergenicity profiling, and selection for food processing traits.

## CONFLICT OF INTEREST

The authors declare no conflicts of interest.

## AUTHOR CONTRIBUTIONS

Yaojie Zheng: conceptualization; methodology; software; validation; formal analysis; investigation; data curation; writing – original draft; visualization. Cynthia Denbow: methodology; software. Matheus Ogando do Granja: writing – original draft. Guillaume Pilot: methodology; writing – review and editing. Zhibo Wang: writing – review and editing. Maria Luciana Rosso: writing – review and editing; project administration. Bo Zhang: conceptualization; validation; resources; writing – review and editing; supervision; project administration; funding acquisition.

## Data Availability

The data that support the findings of this study are available from the corresponding author upon reasonable request.
